# Augmenting the Antifungal Activity of an Oxidizing Agent with Kojic Acid: Control of *Penicillium* Strains Infecting Crops

**DOI:** 10.3390/molecules191118448

**Published:** 2014-11-12

**Authors:** Jong H. Kim, Kathleen L. Chan

**Affiliations:** Foodborne Toxin Detection and Prevention Research Unit, Western Regional Research Center, USDA-ARS, 800 Buchanan St., Albany, CA 94710, USA; E-Mail: kathy.chan@ars.usda.gov

**Keywords:** antifungal, chemosensitization, heat treatment, hydrogen peroxide, kojic acid, mycotoxin, *Penicillium*

## Abstract

Oxidative treatment is one of the strategies for preventing *Penicillium* contamination in crops/foods. The antifungal efficacy of hydrogen peroxide (H_2_O_2_; oxidant) was investigated in *Penicillium* strains by using kojic acid (KA) as a chemosensitizing agent, which can enhance the susceptibility of pathogens to antifungal agents. Co-application of KA with H_2_O_2_ (chemosensitization) resulted in the enhancement of antifungal activity of either compound, when compared to the independent application of each agent alone. Of note, heat enhanced the activity of H_2_O_2_ to a greater extent during chemosensitization, whereby the minimum inhibitory or minimum fungicidal concentrations of H_2_O_2_ was decreased up to 4 or 13 fold, respectively, at 35–45 °C (heat), when compared to that at 28 °C (normal growth temperature). However, heat didn’t increase the antifungal activity of KA, indicating specificity exists between heat and types of antifungals applied. The effect of chemosensitization was also strain-specific, where *P. expansum* (both parental and fludioxonil-resistant mutants) or *P. italicum* 983 exhibited relatively higher susceptibility to the chemosensitization, comparing to other *Penicillium* strains tested. Collectively, chemosensitization can serve as a potent antifungal strategy to lower effective dosages of toxic antifungal substances, such as H_2_O_2_. This can lead to coincidental lowering of environmental and health risks.

## 1. Introduction

**Figure 1:**
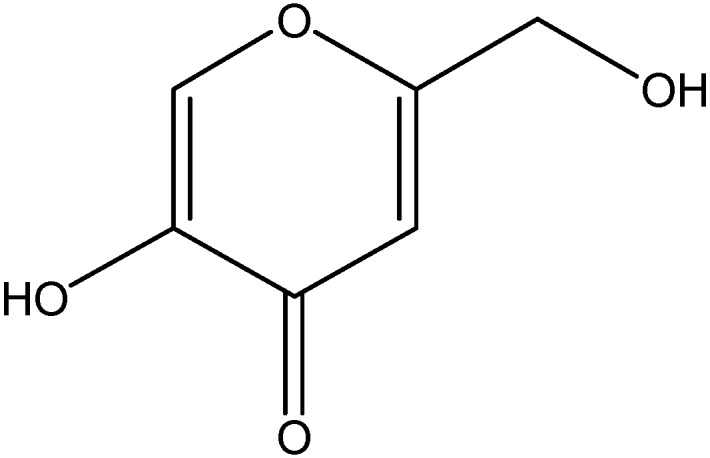
Structure of kojic acid (KA).

Kojic acid (5-hydroxy-2-(hydroxymethyl)-4*H*-pyran-4-one, KA, Figure 1), is a natural compound produced by certain filamentous fungi (*Aspergillus*, *Penicillium*) or *Acetobacter* ([1,2] and references therein)*.* KA is widely used as a food additive, as a depigmenting/skin-whitening agent (via inhibition of tyrosinase, a key enzyme involved in melanogenesis in melanoma and melanocytes), as an antitumor or anti-leishmanial agent, *etc*. [3,4,5,6]. KA can inhibit bacterial/fungal infection [7,8], where KA functions as an enhancer of host immunity [9,10]. For instance, KA stimulates phagocytosis, induces the generation of reactive oxygen species in macrophages, and potentiates phytohemagglutinin-based proliferation of lymphocytes [9,10]. KA also showed a fungistatic antifungal activity against *Cryptococcus neoformans*, a causative agent triggering human cryptococcosis, in which KA inhibited melanin biosynthesis required for fungal infectivity [11].

Certain *Aspergillus* strains, such as *A. flavus* or *A. parasiticus*, produce hepato-carcinogenic aflatoxins (AFs). Recently, the use of atoxigenic (*i.e*., AF non-producing) *Aspergillus* strains as biocontrol agents in agricultural environments [12] has prompted further investigation into their mode of action. It was found that although the atoxigenic strains do not produce AFs, as expected, they do still produce KA [13], which can act as an antagonizing agent against other co-infecting microbes in crops [7]. Therefore, the secondary metabolite KA produced by microorganisms could function as a “biotic” stressor to co-infecting pathogens [7].

The filamentous fungi in the genus *Penicillium* are frequently involved in food contamination or postharvest decay. For example, *P. expansum* is the main producer of the mycotoxin patulin (PAT) that triggers harmful effects on human/animal health [14]. Apples and apple products are the main sources of PAT contamination. PAT is a potential endocrine disruptor, and modulates hormone production [14]. Meanwhile, other *Penicillium* strains, such as *P.* *digitatum* and *P. italicum* are the most devastating citrus pathogens, causing significant economic losses to the industry during post-harvest [15]. Therefore, development of strategies for early intervention of mycotoxin production or fungal contamination in crops or foods is urgently needed.

Various environmental factors, such as fluctuation of temperature, humidity or pH, *etc.*, can be sources of “abiotic” stress to fungal pathogens. Accordingly, abiotic stresses artificially applied could adversely affect the growth of fungi, resulting in the reduction of fungal contamination or postharvest decay. Heat or oxidative treatment is one of the strategies to prevent contamination by *Penicillium* in foods or crops. For example, immersion of pear fruit in heated water (55 °C) controlled *P. expansum*-triggered fruit decay, which also allowed the delay of fruit ripening during storage [16]. Likewise, combined application of oxidizing compounds, such as H_2_O_2_ and sodium hypochlorite (NaClO), resulted in synergistic antifungal effect on *P. expansum*, rendering effective control of postharvest decay in fresh fruits [17].

Co-application of certain types of compounds with conventional antimicrobial drugs/fungicides can enhance the effectiveness of drugs/fungicides through a mechanism termed “chemosensitization” [18,19,20,21]. For example, the combination of the antifungal drug fluconazole (FLC) with various non-antifungal agents, such as traditional Chinese plant extracts, inhibitors of cell signaling (e.g., calcineurin) or heat shock protein 90, *etc.*, increased the susceptibility of the yeast pathogen *Candida albicans* to FLC [20]. Therefore, chemosensitization strategy could lead to (1) lowering dosages of commercial, toxic drugs or antifungal agents required for effective control of pathogens and (2) controlling pathogen resistance to antifungal drugs/agents [22,23,24].

In this study, antifungal chemosensitization is investigated for effective control of pathogenic strains of *Penicillium* (See Table 1) by co-applying KA with H_2_O_2_, which mimics host reactive oxygen species. Levels of compound interactions, at moderate (28 °C) to high temperatures (35, 45 °C), are determined and compared according to the method outlined by the Clinical Laboratory Standards Institute (CLSI) M38-A [25]. Results demonstrate that KA chemosensitizes *Penicillium* strains to H_2_O_2_, lowering the effective dosages of H_2_O_2_ required for control of *Penicillium*. The potency of H_2_O_2_ is greater when *Penicillium* strains are treated with heat (35–45 °C).

**Table 1:** *Penicillium* strains used in this study.

*Penicillium* Strains	Strain Characteristics	Source/Reference
Group A (*P. expansum*):		
*P. expansum* W1	Plant pathogen (Parental strain)	[26]
*P. expansum* FR2	Plant pathogen, Fludioxonil resistant mutant derived from *P. expansum* W1	[26]
*P. expansum* W2	Plant pathogen (Parental strain)	[26]
*P. expansum* FR3	Plant pathogen, Fludioxonil resistant mutant derived from *P. expansum* W2	[26]
Group B (Other *Penicillium* strains):		
*P. glabrum* 766	Plant pathogen	NRRL ^a^
*P. chrysogenum* 824	Fleming’s penicillin-producing strain	NRRL
*P. griseofulvum* 2159	Plant pathogen	NRRL
Group C (Citrus pathogens):		
*P. digitatum* 786	Plant pathogen	NRRL
*P. italicum* 983	Plant pathogen	NRRL

^a^: NRRL, National Center for Agricultural Utilization and Research, USDA-ARS, Peoria, IL, USA.

## 2. Results and Discussion

### 2.1. Susceptibility of P. expansum W1 to High Temperatures

Heat response of *Penicillium* was tested using *P. expansum* W1 (Parental, wild type strain) as a representative strain. *P. expansum* W1 was cultivated on potato dextrose agar (PDA) at high temperatures (35, 45, 55 °C) (1, 2, 3, 4 d), and was then transferred to 28 °C (moderate, normal growth temperature) for growth recovery (for up to 7 d; see Experimental section). For control, W1 was cultivated at 28, 35, 45 and 55 °C for 7 d.

**Figure 2:**
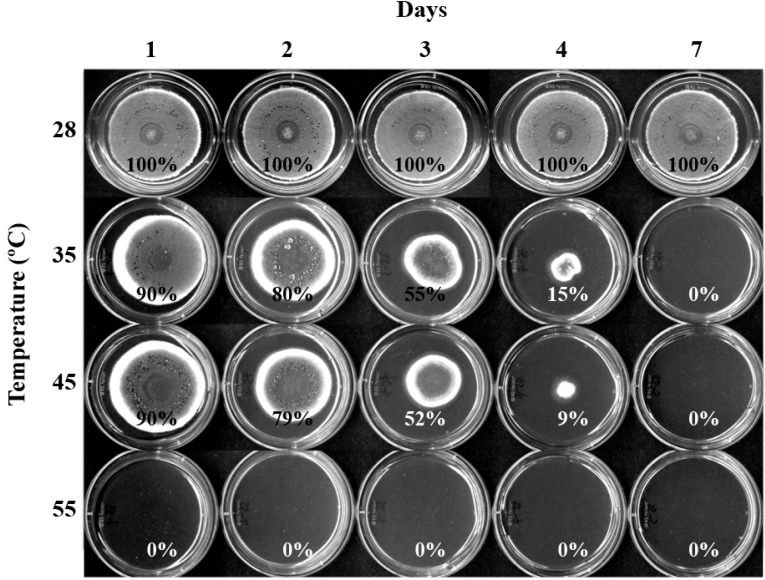
Fungal plate bioassay showing sensitivity of *P. expansum* W1 to high temperatures (35, 45, 55 °C). The % values shown are relative growth rate of W1 compared to that at 28 °C (positive control). SD < 5%.

As shown in Figure 2, *P. expansum* W1 did not grow (namely, no sign of germination) on PDA when it was cultivated solely at 35, 45 or 55 °C for 7 d. However, *P. expansum* W1 treated with heat (35, 45 °C) for 1–4 d could recover growth after cells were transferred to 28 °C (grown up to 7 d). W1 cultivated at 55 °C could not recover growth even after 1 day-heat treatment at 55 °C. Based on this result, moderate (28 °C) to high (35, 45 °C) temperatures, except 55 °C, were chosen for further antifungal investigation in this study.

### 2.2. Susceptibility of Penicillium Strains to Chemosensitization

#### 2.2.1. Effect of Co-Application of KA with H_2_O_2_ on Fungal Growth: at 24 h

At 24 h of CLSI-based fungal cultivation (at 28, 35, 45 °C), *Penicillium* strains didn’t visibly grow in RPMI liquid medium (microtiter plates), and hence, the values of Minimum Inhibitory Concentrations (MICs)/Fractional Inhibitory Concentration Indices (FICIs) could not be determined (Supplementary Tables S1 and S2), whereas, Minimum Fungicidal Concentrations (MFCs)/Fractional Fungicidal Concentration Indices (FFCIs) could be determined when the liquid cultures (200 μL/micotiter plate well) from the respective temperature were transferred onto PDA and cultivated for additional 48 h (at 28 °C; see Experimental section) except *P. digitatum* 786, which was unable to grow on PDA if cultured at 45 °C in microtiter plates (namely, heat sensitive). The values of FFCIs ranged from “additive” (0.5 < FFCI ≤ 1) to “neutral” (1 < FFCI ≤ 2) [27] (Supplementary Table S1 (Average MFC/FFCI values of Group A, B, C strains), Supplementary Table S2 (MFC/FFCI values of individual strains in Group A, B, C)). Despite the absence of calculated “synergistic” interaction, as determined by “additive” or “neutral” interactions during chemosensitization (Supplementary Table S1), there was enhanced antifungal activity of H_2_O_2_ and also KA in most *Penicillium* strains examined at 24 h, which was reflected in lowered MFCs of each compound when combined.

In general, the higher the temperature, the lower the concentration of H_2_O_2_ required for achieving ≥99.9% killing of *Penicillium* strains (*viz*. enhanced antifungal activity of H_2_O_2_ by heat). For example, the average MFCs (MFC_Alone_) of H_2_O_2_ in Group A were 7.0, 2.0 or 0.6 mM at 28, 35 or 45 °C, respectively, thus exhibiting the highest or lowest MFC_Alone_ value at 28 or 45 °C, respectively. When H_2_O_2_ was co-applied with KA (chemosensitization), the average MFCs of H_2_O_2_ were lowered further, resulting in MFC_Combined_ values of 4.0, 1.0 or 0.3 mM at 28, 35 or 45 °C, respectively. Therefore, the values of MFC_Combined_ (chemosensitization) were 1.7 to 2.0 times lower comparing to that of MFC_Alone_ (independent application). Of note, there was up to 13-fold decrease in MFC_Combined_ in Group A at 45 °C (MFC: 0.3 mM) comparing to that at 28 °C (MFC: 4.0 mM).

The effect of chemosensitization was also identified in Group B, where co-application of H_2_O_2_ with KA lowered the MFC values of H_2_O_2_. Consequently, chemosensitization resulted in 1.2 to 1.8 times lower MFC values (MFC_Combined_) of H_2_O_2_ comparing to the independent application of H_2_O_2_, alone. The only exception is *P. griseofulvum* 2159, which showed indifference to chemosensitization (namely, no change in MFC values with KA + H_2_O_2_; FFCI value = 2.0). Comparing to Group A, the average MFCs of H_2_O_2_ for Group B were relatively higher, *viz*. 1.8 to 4.0 times higher for MFC_Alone_ and 2.0 to 4.7 times higher for MFC_Combined_, respectively (Supplementary Table S1**)**. Therefore, results indicated that Group B strains were less susceptible to H_2_O_2_ than the Group A.

In Group C, two citrus pathogens were examined for their responses to H_2_O_2_/KA. The MFC values of H_2_O_2_ in *P. italicum* 983 were 2, 1 or 0.125 mM for MFC_Alone_ (independent application) and 1, 1 or 0.0625 mM for MFC_Combined_ (chemosensitization) at 28, 35 or 45 °C, respectively (Supplementary Table S2). Hence, *P*. *italicum* 983 exhibited similar trends of H_2_O_2_ sensitivity to Group A or B strains, where (1) heat enhanced H_2_O_2_ activity and (2) chemosensitization enhanced the antifungal activity of H_2_O_2_ (Exception: no change in MFC_Combined_ at 35 °C). However, tests with *P. digitatum* 786, the other citrus pathogen, showed that while the MFCs (MFC_Alone_) of H_2_O_2_ were lowered from 4 to 1 mM when temperature was increased from 28 to 35 °C (namely, heat enhancement of H_2_O_2_ activity), this strain was unable to grow at 45 °C (*viz*. higher heat sensitivity than *P. italicum* 983). Moreover, *P. digitatum* 786 showed indifference to KA-mediated chemosensitization, resulting in no change in MFC values when KA was co-applied with H_2_O_2_ (Supplementary Table S2; FFCI = 2.0). Therefore, two citrus pathogens exhibited different responses to the antifungal treatments. Noteworthy is that *P. digitatum* was previously shown to possess a capability to suppress a defense-related H_2_O_2_ production in host tissue [28]. Collectively, results indicated that the effect of antifungal chemosensitization is strain-specific.

Regarding the chemosensitizing agent KA, MFC values of KA were also lowered in most strains when KA was co-applied with H_2_O_2_, where the level of average MFC_Combined_ was 1.3 to 2.3 times lower than MFC_Alone_ depending on types of strains (Supplementary Table S1; See Supplementary Table S2 for exceptions, where FFCI = 2.0). However, unlike in H_2_O_2_, heat did not enhance the activity of KA, suggesting specificity also exists between heat and types of antifungal compounds applied for the enhancement of antifungal activity.

Altogether, at 24 h, KA chemosensitizes most *Penicillium* strains tested, where co-application of KA with H_2_O_2_ resulted in increased antifungal activity of either agents. Furthermore, heat (35, 45 °C) enhanced the activity of H_2_O_2_, while that of KA was vastly unaffected by heat treatment. Regarding the stability of H_2_O_2_, H_2_O_2_ is sensitive to light, pH and/or heat. Especially, heat can induce chemical decomposition of H_2_O_2_ into H_2_O and O_2_. The O_2_ generated can be used for mitochondrial respiration in fungi, while it also means increase of pressure. Therefore, O_2_ level and pressure as well as the level of mitochondrial respiration may be of interest in the future chemosensitization study.

#### 2.2.2. Effect of Co-Application of KA with H_2_O_2_ on Fungal Growth: at 48 h

At 48 h of CLSI-based fungal cultivation, both MICs and MFCs (thus FICI and FFCI values, accordingly) could be determined depending on types of *Penicillium* strains or growth temperatures. For instance, MICs/FICIs could be measured in Group A or C at 28 °C (in microtiter plates), while those values could not be determined at 35 or 45 °C due to incapability of their growth at the higher temperatures. Whereas, MICs/FICIs could be determined in Group B at both 28 and 35 °C (45 °C: no growth), thus showing relatively higher tolerance of Group B to heat (35 °C) comparing to Group A or C in the liquid culture (Table 2, Supplementary Table S3).

The MIC values, namely MIC_Alone_ or MIC_Combined_, of H_2_O_2_ or KA were lowered after chemosensitization, as follows: (1) 2.2 or 3.5 times lowered for H_2_O_2_ or KA, respectively, in Group A (28 °C), (2) 1.5 or 1.7 times lowered for H_2_O_2_ and 1.4 or 2.1 times lowered for KA at 28 or 35 °C, respectively, in Group B, and (3) 2.0 times lowered for both H_2_O_2_ and KA in Group C (28 °C). In all Groups, FICI values ranged from “additive” (0.5 < FICI ≤ 1) to “neutral” (1 < FICI ≤ 2) [27]. Of note, in Group B, there was up to 4-fold decrease in MIC_Combined_ when temperature was increased from 28 °C (MIC: 5.3 mM) to 35 °C (MIC: 1.3 mM). Despite the absence of calculated “synergistic” interaction, as determined by “additive” or “neutral” interactions (Table 2), there was enhanced antifungal activity of H_2_O_2_ and KA in most *Penicillium* strains examined at 48 h*,* which was reflected in lowered MICs of each compound when combined.

**Figure 3:**
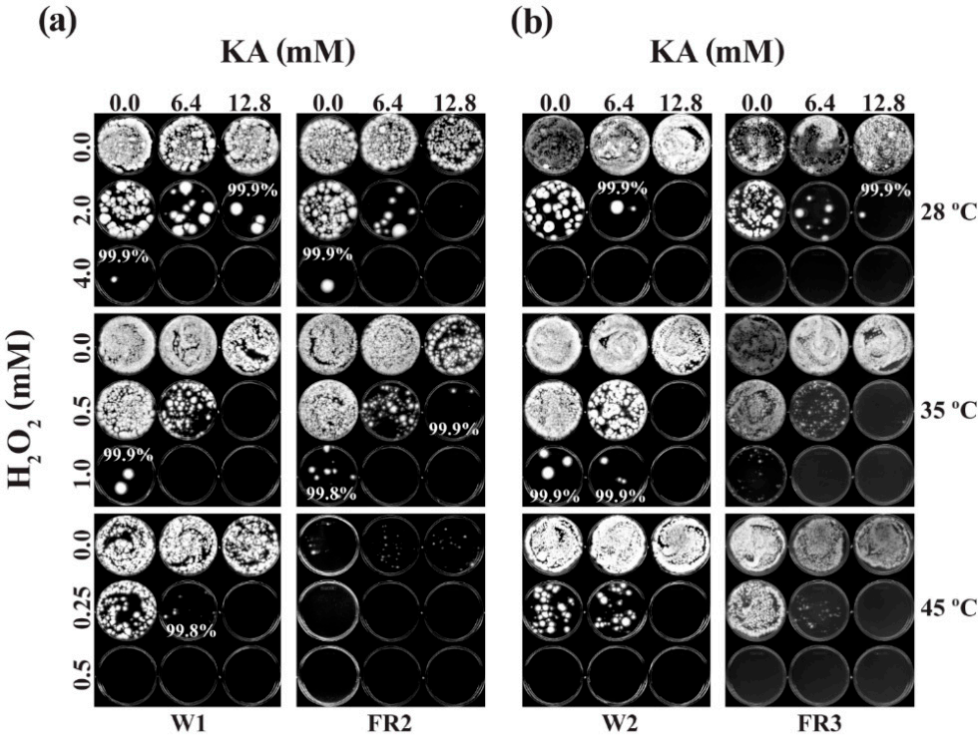
Chemosensitization (KA + H_2_O_2_) test in *P. expansum* wild type and fludioxonil-resistant mutants at moderate (28 °C) to high (35, 45 °C) temperatures. Results shown here are determination of MFCs of antifungal agents (48 h). (**a**) W1 and FR2; (**b**) W2 and FR3.

The values of MFCs/FFCIs could also be determined on PDA for most strains (Group A, B or C) at 28, 35 and 45 °C. Exceptions are *P. glabrum* 766, *P. chrysogenum* 824, *P. italicum* 983 and *P. digitatum* 786, which were unable to grow at 45 °C. Noteworthy is that, when compared to 24 h (See above; Supplementary Tables S1 and S2), 48 h of cultivation of *Penicillium* strains, in general, required lower concentration of H_2_O_2_ to achieve ≥99% fungal death. For example, when Group A strains were co-treated with 12.8 mM of KA (chemosensitization) for 48 h, ≥99.9% fungal death was achieved with 2.0, 0.5 or 0.2 mM of H_2_O_2_ at 28, 35 or 45 °C, respectively (Table 2; See also Figure 3), while similar level of fungal death was achieved with 4.0, 1.0 or 0.3 mM of H_2_O_2_ at 28, 35 or 45 °C, respectively, at 24 h (Supplementary Table S1). Therefore, when compared to 24 h, 1.5 to 2-fold less concentrations of H_2_O_2_ were required to achieve ≥99.9% fungal death at 48 h during chemosensitization. Similar trends were also observed in Group B and C (The only exception was MFCs in Group C at 28 °C, where MFC_Alone_ or MFC_Combined_ was similar to or slightly higher than that of 24 h).

**Table 2:** Antifungal chemosensitization of kojic acid (KA; mM) to hydrogen peroxide (H_2_O_2_; mM) at different temperatures tested against *Penicillium* strains. Summary of CLSI-based microdilution bioassays (Average MIC/FICI and MFC/FFCI values of Group A, B, C strains at 48 h) ^a^.

Group A (*P. expansum* W1, FR2, W2, FR3)							
28 °C							
	**Compounds**	**MIC Alone**	**MIC Combined**	**FICI**	**MFC Alone**	**MFC Combined**	**FFCI**
**Mean**	KA	25.6	7.2	0.7	25.6 ^b^	10.4	0.9
	H_2_O_2_	4.0	1.8		4.0	2.0	
***t*-test **	KA	-	*p* <0.005	-	-	*p* <0.005 ^c^	-
	H_2_O_2_	-	*p* <0.005	-	-	*p* <0.005	-
35 °C							
**Mean**	KA	/	/	/	25.6	12.8	0.8
	H_2_O_2_	/	/		1.5	0.5	
***t*-test **	KA	-	/	-	-	*p* <0.005	-
	H_2_O_2_	-	/	-	-	*p* <0.05	-
45 °C							
**Mean**	KA	/	/	/	25.6	16.0	0.6
	H_2_O_2_	/	/		0.4	0.2	
***t*-test **	KA	-	/	-	-	*p* <0.05	-
	H_2_O_2_	-	/	-	-	*P, insignificant*	-
**Group B (*P. glabrum* 766, *P.chrysogenum* 824, *P.griseofulvum* 2159)**							
28 °C							
Mean	KA	25.6	17.6	1.4	25.6	13.9	1.1
	H_2_O_2_	8.0	5.3		8.0	4.7	
*t*-test	KA	-	*P, insignificant*	-	-	*P, insignificant*	-
	H_2_O_2_	-	*P, insignificant*	-	-	*P, insignificant*	-
35 °C							
Mean	KA	25.6	11.7	1.0	25.6	19.2	1.4
	H_2_O_2_	2.3	1.3		4.0	2.7	
*t*-test	KA	-	*P, insignificant P, insignificant*	-	-	*P, insignificant*	-
	H_2_O_2_				-	*P, insignificant*	-
45 °C							
Mean	KA	/	/	/	/	/	/, ND ^d^
	H_2_O_2_	/	/		/	/	
t-test	KA	-	/	-	-	/	-
	H_2_O_2_	-	/	-	-	/	-
**Group C (*P. italicum* 983, *P. digitatum* 786)**							
28 °C							
Mean	KA	25.6	12.8	1.0	25.6	25.6	2.0
	H_2_O_2_	3.0	1.5		3.0	3.0	
t-test	KA	-	ND ^e^	-	-	ND ^e^	-
	H_2_O_2_	-	ND ^e^	-	-	ND ^e^	-
35 °C							
Mean	KA	/	/	/	25.6	19.2	1.4
	H_2_O_2_	/	/		0.6	0.4	
t-test	KA	-	/	-	-	ND ^e^	-
	H_2_O_2_		/		-	ND ^e^	-
45 °C							
Mean	KA	/	/	/	/	/	/, ND ^f^
	H_2_O_2_	/	/		/	/	
t-test	KA	-	/	-	-	/	-
	H_2_O_2_		/		-	/	-

^a^ MIC: Minimum inhibitory concentration, MFC: Minimum fungicidal concentration, FICI: Fractional Inhibitory Concentration Indices, FFCI: Fractional Fungicidal Concentration Indices; ^b^ KA was tested up to 12.8 mM. For calculation purpose, 25.6 mM (doubling of 12.8 mM) was used; ^c^ Student’s *t*-test for paired data (combined, *i.e.*, chemosensitization) was *vs.* mean MIC or MFC of each compound (alone, *i.e.*, no chemosensitization) determined in strains; ^d^ ND, Not determined (No cell growth except *P. griseofulvum* 2159, for which FFCI is neutral); ^e^ ND, Not determined (Few data); ^f^ ND, Not determined (No growth of Group C strains).

As observed in 24 h, the higher the temperature, the lower the concentration of H_2_O_2_ needed for achieving ≥99.9% killing of *Penicillium* strains at 48 h. For example, the average MFC_Alone_ values of H_2_O_2_ in Group A at 48 h were 4.0, 1.5 or 0.4 mM at 28, 35 or 45 °C, respectively, indicating 10 times lower concentration of H_2_O_2_ (45 °C) was required to achieve ≥99.9% fungal death, when compared to 28 °C. With the chemosensitization, MFC_Combined_ values of H_2_O_2_ were lowered further to 2.0, 0.5 or 0.2 mM at 28, 35 or 45 °C, respectively, thus showing that 10 times less H_2_O_2_ was needed to achieve ≥99.9% fungal death at 45 °C, when compared to 28 °C. Similar trends in the heat enhancement of H_2_O_2_ activity were also found in Groups B and C during chemosensitization. As observed in 24 h, the average MFCs of H_2_O_2_ in Group B (both MFC_Alone_ and MFC_Combined_) were relatively higher than that in Group A (namely, 2.0 to 5.4 times higher), thus confirming further that Group B was less susceptible to H_2_O_2_ than Group A.

Interestingly, although Group B exhibited higher tolerance to heat (at 35 °C) comparing to Group A or C (See above; microtiter plate liquid bioassay), two strains in Group B, *P. glabrum* 766 and *P.* *chrysogenum* 824, were unable to recover their growth on PDA at the elevated temperature, *viz*. 45 °C (thus cannot determine their MFCs/FFCIs). However, Group A strains could still recover their growth at 45 °C. Thus, results indicated differential range of optimum growth temperatures for each fungus tested.

Regarding the chemosensitizing agent KA, MFCs of KA were also lowered in most strains when KA was co-applied with H_2_O_2_, where the level of MFC_Combined_ of KA (chemosensitization) was 1.3 to 2.4 times lower than MFC_Alone_ (independent application), depending on types of strains (See Supplementary Table S3 for exceptions, where FICI or FFCI = 2.0). However, at 45 °C, MIC/MFC values of either H_2_O_2_ or KA in Group B or C couldn’t be determined since most strains in these Groups were unable to grow.

Collectively, at 48 h, KA chemosensitizes most *Penicillium* strains tested, where co-application of KA with H_2_O_2_ resulted in increased antifungal activity of either agents. As observed in 24 h, heat (35, 45 °C) enhanced the activity of H_2_O_2_, while that of KA was vastly unaffected by heat treatment. Therefore, results proved further that specificity exists between heat and types of antifungals applied. Also, effect of chemosensitization was strain-specific, where *P. expansum* (both parental and fludioxonil-resistant mutant strains) or *P. italicum* 983 exhibited relatively higher susceptibility to H_2_O_2_, comparing to other *Penicillium* strains. Considering KA can be oxidized by H_2_O_2_ under heating conditions, the oxidized KA might also possess an antifungal property. Determination of the precise amount of KA by using HPLC, *etc.*, warrants future study.

#### 2.2.3. Effect of High Temperatures on the Growth of Fludioxonil-Resistant Mutants

The fludioxonil-resistant mutant *P. expansum* FR2 showed hypersensitivity to heat (45 °C) comparing to other *P. expansum* strains (W1, W2, FR3). As shown in Figure 3, *P. expansum* FR2 barely grew at 45 °C, where very tiny colonies appeared on PDA even without H_2_O_2_ or KA treatment. If FR2 was treated with H_2_O_2_ (as low as 0.25 mM) at 45 °C, colony growth was completely inhibited (namely, no signs of germination). Whereas, the impact of KA on the growth of FR2 at 45 °C was negligible (See Figure 3). Therefore, results indicated that fludioxonil resistance and heat sensitivity in FR2 are co-segregating traits, where the mutation responsible for fungicide resistance might also affect cellular fitness and/or mitochondrial function [29] in the presence of heat stress. Of note, a similar interrelationship between fungicide resistance and fungal sensitivity to high temperatures was previously identified in another fungal plant pathogen, *Monilinia fructicola* [30]*.*

**Figure 4:**
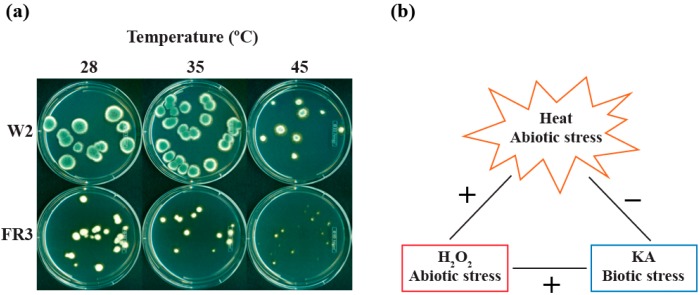
(**a**) Reduced growth of *P. expansum* FR3, a fludioxonil-resistant mutant, comparing to W2, a parental strain; (**b**) Diagram showing the interaction characteristics between heat, KA and H_2_O_2_. +: positive interaction (*i.e*., enhancement of antifungal activities when combined), −: no interaction (*i.e*., no enhancement of antifungal activities when combined).

The other fludioxonil-resistant mutant, *P. expansum* FR3, did not exhibit similar type of heat sensitivity as observed in FR2 (Figure 3). However, in a parallel study, the colony growth of FR3 was noticeably reduced compared to that of *P. expansum* W2 (parental strain) (Figure 4a). For instance, both *P. expansum* W2 and FR3 exhibited gradual reduction in colony growth on PDA when the temperature was increased from 28 °C to 45 °C (Figure 4a). However, the colony size of *P. expansum* FR3 mutant was much smaller than that of W2 (parental), even at the normal growth temperature (28 °C). Moreover, the color of FR3 colonies remained pale at all conditions, while that of W2 was blue, indicating cellular physiology or a process, such as secondary metabolism responsible for pigment development, in FR3 is also impaired. Similar type of interrelationship between fungicide resistance and reduced fungal growth was previously identified in other fungi [31].

Altogether, results from two fludioxonil resistant mutants (FR2, FR3) showed that one or more traits are co-segregated with fungicide resistance. Previous study showed that certain fungi with mutations in genes responsible for signal transduction of environmental stress, such as Mitogen-Activated Protein Kinase (MAPK) signaling pathway, could develop fludioxonil resistance [32]. Coinciding with the fludioxonil resistance was the increased sensitivity of this MAPK mutant to high osmotic stress, thus exhibiting co-segregation of two different traits. Alternatively, results suggested that vulnerable or susceptible targets for fungal control could be identifiable in fungicide resistant mutants, such as heat/stress sensitivity or impaired growth, *etc.*, as determined in this study. Precise characterization of the links between heat susceptibility/reduced growth and fludioxonil resistance, identified in FR2 and FR3, warrants future study. Summary of agent interactions, between biotic and abiotic stressors, is described in Figure 4b.

## 3. Experimental Section

### 3.1. Fungal Strains and Culture Conditions

Fungal strains used in this study are summarized in Table 1. *Penicillium* strains were cultured on potato dextrose agar (PDA) at 28 °C, except when otherwise noted in the text (see also below).

### 3.2. Chemicals

Antifungal compounds (kojic acid (KA) (Figure 1), hydrogen peroxide (H_2_O_2_)) were procured from Sigma Co. (St. Louis, MO, USA). KA was dissolved in dimethylsulfoxide (DMSO; absolute DMSO amount: <2% in media) before incorporation into culture media. Throughout this study, control plates (No treatment) contained DMSO at levels equivalent to that of cohorts receiving antifungal agents, within the same set of experiments. H_2_O_2_ was diluted in sterile water before incorporation into culture media.

### 3.3. Antifungal Bioassay

#### 3.3.1. Growth Recovery Test in *Penicillium* Strain Treated with High Temperatures: Agar Plate-Bioassay

Agar plate-based bioassay was performed to evaluate susceptibility of *Penicillium expansum* W1 to high temperatures. First, fungal conidia (5 × 10^3^) were spotted on PDA (triplicate PDA plates per each temperature), and were initially incubated at three different temperatures (35, 45, 55 °C). Triplicate PDA plates were then removed from each temperature (35, 45 or 55 °C) at day 1, 2, 3, and 4, and were transferred to 28 °C for additional 6, 5, 4, and 3 d of growth, respectively, resulting in a total of 7 d of incubation for each treatment (e.g., 1 d growth at 45 °C + 6 d growth at 28 °C = Total 7 d growth, 2 d growth at 45 °C + 5 d growth at 28 °C = Total 7 d growth, 3 d growth at 45 °C + 4 d growth at 28 °C = Total 7 d growth, 4 d growth at 45 °C + 3 d growth at 28 °C = Total 7 d growth). For controls, *Penicillium* strains were grown solely at 28 °C or respective temperature (35, 45, 55 °C) for 7 d. The level of growth recovery at 28 °C was evaluated based on fungal radial growth, which was compared to that determined at 28 °C.

#### 3.3.2. Microtiter Plate (Microdilution) Liquid Bioassay

To determine the precise level of chemosensitizing activity of KA (0.1, 0.2, 0.4, 0.8, 1.6, 3.2, 6.4, 12.8 mM) to H_2_O_2_ (0.25, 0.5, 1, 2, 4, 8, 16 mM) in the strains of *Penicillium*, checkerboard bioassays (triplicate) (0.4 × 10^4^–5 × 10^4^ CFU/mL) were performed in microtiter wells (at 28, 35, 45 °C) using a broth microdilution method (in RPMI 1640 medium; Sigma Co., St. Louis, MO, USA), according to protocols outlined by the Clinical and Laboratory Standards Institute (CLSI) M38-A [25]. RPMI 1640 medium was supplemented with 0.03% L-glutamine and buffered with 0.165 mM 3-(N-morpholino) propanesulfonic acid. Minimum Inhibitory Concentrations (MICs), lowest concentration of agents showing no visible fungal growth in microtiter wells (200 μL per well), were assessed after 48 h. Minimum Fungicidal Concentrations (MFCs), lowest concentration of agents showing ≥99.9% fungal death, were determined following completion of MIC assays (28, 35, 45 °C) by spreading entire volumes of microtiter wells (200 μL) onto individual PDA plates, and culturing for another 48 h (at 28 °C). Compound interactions, Fractional Inhibitory Concentration Indices (FICIs) and Fractional Fungicidal Concentration Indices (FFCI), were calculated as follows: FICI or FFCI = (MIC or MFC of compound A in combination with compound B/MIC or MFC of compound A, alone) + (MIC or MFC of compound B in combination with compound A/MIC or MFC of compound B, alone). Levels and types of compound interactions between antifungal agents (H_2_O_2_ and KA) were defined as: synergistic (FICI ≤ 0.5), additive (0.5 < FICI ≤ 1), neutral (1 < FICI ≤ 2) or antagonistic (2 < FICI) [27]. If preferred, the Odds’ [33] methodology can be substituted in parallel calculations of compound interactions.

### 3.4. Statistical Analysis

Statistical analysis (student’s *t*-test) was performed based on “Statistics to use” [34], where *p* < 0.05 was considered significant.

## 4. Conclusions

In this study, levels of interactions between biotic (KA) and abiotic (H_2_O_2_) stressors were determined for the enhancement of antifungal efficacy as follows: With chemosensitization (KA + H_2_O_2_), (1) MIC or MFC of KA or H_2_O_2_ was lowered during chemosensitization depending on types of strains or culture conditions (See Table 3 for summary). Since KA induces the generation of reactive oxygen species in cells, such as macrophages (during phagocytosis) [9,10], elevated oxidative stress occurred during KA-mediated chemosensitization in the presence of H_2_O_2_ (which also mimics host reactive oxygen species) may be the possible mechanism of enhanced activity of the combinational treatment (KA + H_2_O_2_).

**Table 3:** Responses of *Penicillium* strains to chemosensitization (Summary). Data shown are comparison of MICs or MFCs of H_2_O_2_ (antifungal oxidant) or KA (chemosensitizing agent) treated alone or in combination at different temperatures (MICs at 24 h are not determined due to no growth of all strains examined).

		H_2_O_2_			KA		
Strains	Treatment	28 °C	35 °C	45 °C	28 °C	35 °C	45 °C
**MICs, 48 h**							
Group A	Alone	4.0	ND ^a^	ND ^a^	25.6	ND ^a^	ND ^a^
	Combined	1.8	ND ^a^	ND ^a^	7.2	ND ^a^	ND ^a^
Group B	Alone	8.0	2.3	ND ^a^	25.6	25.6	ND ^a^
	Combined	5.3	1.3	ND ^a^	17.6	11.7	ND ^a^
Group C	Alone	3.0	ND ^a^	ND ^a^	25.6	ND ^a^	ND ^a^
	Combined	1.5	ND ^a^	ND ^a^	12.8	ND ^a^	ND ^a^
**MFCs, 24 h**							
Group A	Alone	7.0	2.0	0.6	25.6	25.6	25.6
	Combined	4.0	1.0	0.3	11.2	12.8	11.2
Group B	Alone	14.7	8.0	1.1	25.6	25.6	25.6
	Combined	8.0	4.7	0.9	13.9	17.1	17.1
Group C	Alone	3.0	1.0	ND ^b^	25.6	25.6	ND ^b^
	Combined	2.5	1.0	ND ^b^	19.2	25.6	ND ^b^
**MFCs, 48 h**							
Group A	Alone	4.0	1.5	0.4	25.6	25.6	25.6
	Combined	2.0	0.5	0.2	10.4	12.8	16.0
Group B	Alone	8.0	4.0	ND ^c^	25.6	25.6	ND ^c^
	Combined	4.7	2.7	ND ^c^	13.9	19.2	ND ^c^
Group C	Alone	3.0	0.6	ND ^d^	25.6	25.6	ND ^d^
	Combined	3.0	0.4	ND ^d^	25.6	19.2	ND ^d^

^a^ ND: Not determined (No growth of strains); ^b^ ND: Not determined (No growth of *P. digitatum* 786); ^c^ ND: Not determined (No cell growth except *P. griseofulvum* 2159, for which FFCI is neutral); ^d^ ND: Not determined (No growth of Group C strains).

Considering KA affects the cellular nitric oxide metabolism [35], it is possible that nitrosative stress, such as peroxynitrite, a toxic free radical, may also be linked to the chemosensitization; (2) Most *P. expansum* strains (Group A) tested were sensitive to KA-mediated chemosensitization. In Group B, *P. glabrum* 766 was sensitive to chemosensitization in almost all conditions tested, while *P. griseofulvum* 2159 was mostly insensitive to chemosensitization. *P. chrysogenum* 824 was sensitive to chemosensitization at 24 h, while it was insensitive at 48 h; (3) In Group C, the citrus pathogens, the effect of chemosensitization was dependent upon cultivation time and temperatures, where *P. digitatum* 786 showed less sensitivity to the chemosensitization comparing to *P. italicum* 983. Altogether, the effectiveness of KA-mediated chemosensitization with H_2_O_2_ was fungal strain-specific. We speculate that different levels of antioxidant defense efficiency, metabolism or responses to nitrosative stress, *etc.*, in different *Penicillium* strains (*i.e*., Groups A, B and C) may trigger the differential responsiveness of fungi to the chemosensitization. The fact that KA is produced by different *Penicillium* strains ([1] and references therein) may also explain why several *Penicillium* strains are not that much sensitive to KA and H_2_O_2_. Comparison of the production of KA by each analyzed *Penicillium* strain warrants future study; (4) Comparing to 24 h, 48 h of cultivation of *Penicillium* strains, in general, required lower concentration of H_2_O_2_ to achieve ≥99% fungal death. Results indicated that H_2_O_2_ effectively damaged the cellular integrity of *Penicillium* strains, which may result in the inhibition of cell division cycle as well as antioxidant defense and metabolism in fungi.

The effect of temperatures during chemosensitization is determined as follows: (1) The higher the temperature, the lower the concentration of H_2_O_2_ required for achieving ≥99.9% fungal death, indicating heat and H_2_O_2_ synergize each other for the enhancement of antifungal activity. However, heat did not enhance the activity of KA, indicating specificity also exists between heat and types of antifungal compounds applied for the antifungal efficacy; (2) *P. expansum* FR2, fludioxonil-resistant mutant, showed hypersensitivity to heat (45 °C), while the colony growth of *P. expansum* FR3, the other fludioxonil-resistant mutant, was severely reduced comparing to its parental strain at all temperatures tested. Results indicated that susceptible/sensitive targets for fungal control might be identifiable in such mutants, which is coincided with the mutation involved in fungicide resistance.

In conclusion, KA, a safe, natural compound, possesses a potential to serve as an antifungal chemosensitizing agent in combination with oxidative stressor(s). This potential appears to be greatest with *P. expansum* strains or *P. italicum* 983. Chemosensitization can lower effective dosages of toxic antifungal substances, such as H_2_O_2_, leading to coincidental lowering of environmental and health risks. Antifungal efficacy of H_2_O_2_ was greater when *Penicillium* strains were treated with heat (35 °C, 45 °C). Considering that much higher temperatures (e.g., 55 °C as shown in the Introduction section) are conventionally used for fungal control in crops, the temperatures defined as heat in this study (35, 45 °C), especially during chemosensitization, are markedly lower, and thus could reduce crop damage associated with heat treatment. The use of safe chemosensitizing agents, such as KA, that debilitate fungal pathogens may be a viable approach to control agro/food fungal pathogens.

## Supplementary Materials

Supplementary materials can be accessed at: http://www.mdpi.com/1420-3049/19/11/18448/s1.

## References

[1] Liu X., Xia W., Jiang Q., Xu Y., Yu P. (2014). Synthesis, characterization, and antimicrobial activity of kojic acid grafted chitosan oligosaccharide. J. Agric. Food Chem..

[2] Rodrigues A.P.D., Farias L.H.S., Carvalho A.S.C., Santos A.S., do Nascimento J.L.M., Silva E.O. (2014). A novel function for kojic acid, a secondary metabolite from *Aspergillus* fungi, as antileishmanial agent. PLoS One.

[3] Bentley R. (2006). From miso, sake and shoyu to cosmetics: A century of science for kojic acid. Nat. Prod. Rep..

[4] Chang T.S. (2009). An updated review of tyrosinase inhibitors. Int. J. Mol. Sci..

[5] Lajis A.F., Hamid M., Ariff A.B. (2012). Depigmenting effect of kojic acid esters in hyperpigmented B16F1 melanoma cells. J. Biomed. Biotechnol..

[6] Leyden J.J., Shergill B., Micali G., Downie J., Wallo W. (2011). Natural options for the management of hyperpigmentation. J. Eur. Acad. Dermatol. Venereol..

[7] Bracarense A.A., Takahashi J.A. (2014). Modulation of antimicrobial metabolites production by the fungus *Aspergillus parasiticus*. Braz. J. Microbiol..

[8] Novotny L., Rauko P., Abdel-Hamid M., Vachalkova A. (1999). Kojic acid—A new leading molecule for a preparation of compounds with an anti-neoplastic potential. Neoplasma.

[9] Niwa Y., Akamatsu H. (1991). Kojic acid scavenges free radicals while potentiating leukocyte functions including free radical generation. Inflammation.

[10] Rodrigues A.P., Carvalho A.S., Santos A.S., Alves C.N., do Nascimento J.L., Silva E.O. (2011). Kojic acid, a secondary metabolite from *Aspergillus* sp., acts as an inducer of macrophage activation. Cell Biol. Int..

[11] Chee H.Y., Lee E.H. (2003). Fungistatic activity of kojic acid against human pathogenic fungi and inhibition of melanin production in *Cryptococcus neoformans*. Mycobiology.

[12] Ehrlich K.C., Cotty P.J. (2004). An isolate of *Aspergillus flavus* used to reduce aflatoxin contamination in cottonseed has a defective polyketide synthase gene. Appl. Microbiol. Biotechnol..

[13] Kim J.H., Mahoney N., Chan K.L., Campbell B.C., Haff R.P., Stanker L.H. (2014). Use of benzo analogs to enhance antimycotic activity of kresoxim methyl for control of aflatoxigenic fungal pathogens. Front. Microbiol..

[14] Frizzell C., Elliott C.T., Connolly L. (2014). Effects of the mycotoxin patulin at the level of nuclear receptor transcriptional activity and steroidogenesis *in vitro*. Toxicol. Lett..

[15] Vilanova L., Viñas I., Torres R., Usall J., Jauset A.M., Teixido N. (2012). Infection capacities in the orange-pathogen relationship: Compatible (*Penicillium digitatum*) and incompatible (*Penicillium expansum*) interactions. Food Microbiol..

[16] Dore A., Molinu M.G., Venditti T., D’Hallewin G. (2010). Immersion of “Coscia” pear fruit in water at 55 degrees C for 60 sec controls *Penicillium expansum* decay and delays ripening during short storage. Commun. Agric. Appl. Biol. Sci..

[17] Cerioni L., Lazarte Mde L., Villegas J.M., Rodriguez-Montelongo L., Volentini S.I. (2013). Inhibition of *Penicillium expansum* by an oxidative treatment. Food Microbiol..

[18] Campbell B.C., Chan K.L., Kim J.H. (2012). Chemosensitization as a means to augment commercial antifungal agents. Front. Microbiol..

[19] Lavigne J.P., Brunel J.M., Chevalier J., Pages J.M. (2010). Squalamine, an original chemosensitizer to combat antibiotic-resistant gram-negative bacteria. J. Antimicrob. Chemother..

[20] Liu S., Hou Y., Chen X., Gao Y., Li H., Sun S. (2014). Combination of fluconazole with non-antifungal agents: A promising approach to cope with resistant *Candida albicans* infections and insight into new antifungal agent discovery. Int. J. Antimicrob. Agents.

[21] Niimi K., Harding D.R., Parshot R., King A., Lun D.J., Decottignies A., Niimi M., Lin S., Cannon R.D., Goffeau A. (2004). Chemosensitization of fluconazole resistance in *Saccharomyces cerevisiae* and pathogenic fungi by a D-octapeptide derivative. Antimicrob. Agents Chemother..

[22] Kim J.H., Chan K.L., Mahoney N., Campbell B.C. (2011). Antifungal activity of redox-active benzaldehydes that target cellular antioxidation. Ann. Clin. Microbiol. Antimicrob..

[23] Musiol R., Mrozek-Wilczkiewicz A., Polanski J. (2014). Synergy against fungal pathogens: Working together is better than working alone. Curr. Med. Chem..

[24] Veri A., Cowen L.E. (2014). Progress and prospects for targeting Hsp90 to treat fungal infections. Parasitology.

[25] Clinical and Laboratory Standards Institute (CLSI) (2008). Reference Method for Broth dilution Antifungal Susceptibility Testing of Filamentous Fungi: Approved Standard.

[26] Li H.X., Xiao C.L. (2008). Characterization of fludioxonil-resistant and pyrimethanil-resistant phenotypes of *Penicillium expansum* from apple. Phytopathology.

[27] Isenberg H.D. (1992). Clinical Microbiology Procedures Handbook.

[28] Macarisin D., Cohen L., Eick A., Rafael G., Belausov E., Wisniewski M., Droby S. (2007). *Penicillium digitatum* suppresses production of hydrogen peroxide in host tissue during infection of citrus fruit. Phytopathology.

[29] Chatre L., Ricchetti M. (2014). Are mitochondria the Achilles’ heel of the Kingdom Fungi?. Curr. Opin. Microbiol..

[30] Ma Z., Yoshimura M.A., Michailides T.J. (2003). Identification and characterization of benzimidazole resistance in *Monilinia fructicola* from stone fruit orchards in California. Appl. Environ. Microbiol..

[31] Jayasinghe C.K., Fernando T.H. (1998). Growth at different temperatures and on fungicide amended media: Two characteristics to distinguish *Colletotrichum* species pathogenic to rubber. Mycopathologia.

[32] Kojima K., Takano Y., Yoshimi A., Tanaka C., Kikuchi T., Okuno T. (2004). Fungicide activity through activation of a fungal signalling pathway. Mol. Microbiol..

[33] Odds F.C. (2003). Synergy, antagonism, and what the chequerboard puts between them. J. Antimicrob. Chemother..

[34] Kirkman T.W. Statistics to Use. http://www.physics.csbsju.edu/stats/.

[35] Dung T.T., Kim S.C., Yoo B.C., Sung G.H., Yang W.S., Kim H.G., Park J.G., Rhee M.H., Park K.W., Yoon K. (2014). (5-Hydroxy-4-oxo-4H-pyran-2-yl)methyl 6-hydroxynaphthalene-2-carboxylate, a kojic acid derivative, inhibits inflammatory mediator production via the suppression of Syk/Src and NF-κB activation. Int. Immunopharmacol..

