# Supplementary material for: Augmenting the Antifungal Activity of an Oxidizing Agent with Kojic Acid: Control of Penicillium Strains Infecting Crops
Source: Molecules. 2014 Nov 12;19(11):18448–64. doi: 10.3390/molecules191118448 (PMC6271881; doi:10.3390/molecules191118448)
Supplement: Supplementary file 1 [file molecules-19-18448-s001.pdf]

Supplementary Materials

Table S1. Antifungal chemosensitization of kojic acid (KA; mM) to hydrogen peroxide (H_2O_2; mM) at different temperatures tested against *Penicillium* strains. Summary of CLSI-based microdilution bioassays (Average MFC/FFCI values of Group A, B, C strains at 24 h) (MICs at 24 h are not determined due to no growth of all strains examined) [a].

	Compounds	MFC Alone	MFC Combined	FFCI
Group A (*P. expansum* W1, FR2, W2, FR3)				
28 °C				
**Mean**	KA	25.6 [b]	11.2	1.0
	H_2O_2	7.0	4.0	
***t*-test**	KA	-	$P < 0.005$ [c]	-
	H_2O_2	-	$P < 0.05$	-
35 °C				
**Mean**	KA	25.6	12.8	1.0
	H_2O_2	2.0	1.0	
***t*-test**	KA	-	$P < 0.005$	-
	H_2O_2	-	$P < 0.005$	-
45 °C				
**Mean**	KA	25.6	11.2	0.9
	H_2O_2	0.6	0.3	
***t*-test**	KA	-	$P < 0.005$	-
	H_2O_2	-	$P < 0.1$	-
Group B (*P. glabrum* 766, *P. chrysogenum* 824, *P. griseofulvum* 2159)				
28 °C				
Mean	KA	25.6	13.9	1.1
	H_2O_2	14.7	8.0	
t-test	KA	-	*P*, insignificant	-
	H_2O_2	-	*P*, insignificant	-
35 °C				
Mean	KA	25.6	17.1	1.3
	H_2O_2	8.0	4.7	
t-test	KA	-	*P*, insignificant	-
	H_2O_2	-	*P*, insignificant	-
45 °C				
Mean	KA	25.6	17.1	1.5
	H_2O_2	1.1	0.9	
t-test	KA	-	*P*, insignificant	-
	H_2O_2	-	*P*, insignificant	-
Group C (*P. italicum* 983, *P. digitatum* 786):				
28 °C				
Mean	KA	25.6	19.2	0.8
	H_2O_2	3.0	2.5	
t-test	KA	-	ND [d]	-
	H_2O_2	-	ND [d]	-

Table S1. *Cont.*

	Compounds	MFC Alone	MFC Combined	FFCI
		35 °C		
Mean	KA	25.6	25.6	2.0
	H_2O_2	1.0	1.0	
t-test	KA	-	ND [d]	-
	H_2O_2	-	ND [d]	-
		45 °C		
Mean	KA	/	/	ND [e]
	H_2O_2	/	/	
t-test	KA	-	/	-
	H_2O_2	-	/	-

[a] MIC: Minimum inhibitory concentration, MFC: Minimum fungicidal concentration, FICI: Fractional Inhibitory Concentration Indices, FFCI: Fractional Fungicidal Concentration Indices. [b] KA was tested up to 12.8 mM. For calculation purpose, 25.6 mM (doubling of 12.8 mM) was used. [c] Student's *t*-test for paired data (combined, *i.e.*, chemosensitization) was *vs*. mean MFC of each compound (alone, *i.e.*, no chemosensitization) determined in strains. [d] ND, Not determined (Few data). [e] ND, Not determined (No growth of *P. digitatum* 786).

Table S2. Antifungal chemosensitization of kojic acid (KA; mM) to hydrogen peroxide (H_2O_2; mM) at different temperatures, tested against strains of *Penicillium*: summary of CLSI-based microdilution bioassays (24 h) [a].

28 °C	Compounds	MIC Alone	MIC Combined	FICI	MFC Alone	MFC Combined	FFCI
			Group A				
P. expansum W1	KA	/	/	/	25.6 [b]	12.8	1.0
	H_2O_2	/	/		8	4	
P. expansum FR2	KA	/	/	/	25.6	6.4	0.8
	H_2O_2	/	/		8	4	
P. expansum W2	KA	/	/	/	25.6	12.8	1.5
	H_2O_2	/	/		4	4	
P. expansum FR3	KA	/	/	/	25.6	12.8	1.0
	H_2O_2	/	/		8	4	
			Group B				
P. glabrum 766	KA	/	/	/	25.6	12.8	1.0
	H_2O_2	/	/		32	16	
P. chrysogenum 824	KA	/	/	/	25.6	3.2	0.6
	H_2O_2	/	/		8	4	
P. griseofulvum 2159	KA	/	/	/	25.6	25.6	2.0
	H_2O_2	/	/		4	4	
			Group C				
P. italicum 983	KA	/	/	/	25.6	12.8	1.0
	H_2O_2	/	/		2	1	
P. digitatum 786	KA	/	/	/	25.6	25.6	2.0
	H_2O_2	/	/		4	4	

Table S2. *Cont.*

35 °C	Compounds	MIC Alone	MIC Combined	FICI	MFC Alone	MFC Combined	FFCI
Group A							
P. expansum W1	KA<br>H_2O_2	/<br>/	/<br>/	/	25.6<br>2	12.8<br>1	1.0
P. expansum FR2	KA<br>H_2O_2	/<br>/	/<br>/	/	25.6<br>2	12.8<br>1	1.0
P. expansum W2	KA<br>H_2O_2	/<br>/	/<br>/	/	25.6<br>2	12.8<br>1	1.0
P. expansum FR3	KA<br>H_2O_2	/<br>/	/<br>/	/	25.6<br>2	12.8<br>1	1.0
Group B							
P. glabrum 766	KA<br>H_2O_2	/<br>/	/<br>/	/	25.6<br>16	12.8<br>8	1.0
P. chrysogenum 824	KA<br>H_2O_2	/<br>/	/<br>/	/	25.6<br>4	12.8<br>2	1.0
P. griseofulvum 2159	KA<br>H_2O_2	/<br>/	/<br>/	/	25.6<br>4	25.6<br>4	2.0
Group C							
P. italicum 983	KA<br>H_2O_2	/<br>/	/<br>/	/	25.6<br>1	25.6<br>1	2.0
P. digitatum 786	KA<br>H_2O_2	/<br>/	/<br>/	/	25.6<br>1	25.6<br>1	2.0
**45 °C**	**Compounds**	**MIC Alone**	**MIC Combined**	**FICI**	**MFC Alone**	**MFC Combined**	**FFCI**
Group A							
P. expansum W1	KA<br>H_2O_2	/<br>/	/<br>/	/	25.6<br>0.5	12.8<br>0.25	1.0
P. expansum FR2	KA<br>H_2O_2	/<br>/	/<br>/	/	25.6<br>0.5	6.4<br>0.25	0.8
P. expansum W2	KA<br>H_2O_2	/<br>/	/<br>/	/	25.6<br>0.5	12.8<br>0.25	1.0
P. expansum FR3	KA<br>H_2O_2	/<br>/	/<br>/	/	25.6<br>1	12.8<br>0.5	1.0
Group B							
P. glabrum 766	KA<br>H_2O_2	/<br>/	/<br>/	/	25.6<br>0.25	12.8<br>0.125	1.0
P. chrysogenum 824	KA<br>H_2O_2	/<br>/	/<br>/	/	25.6<br>1	12.8<br>0.5	1.0
P. griseofulvum 2159	KA<br>H_2O_2	/<br>/	/<br>/	/	25.6<br>2	25.6<br>2	2.0
Group C							
P. italicum 983	KA<br>H_2O_2	/<br>/	/<br>/	/	25.6<br>0.125	12.8<br>0.0625	1.0
P. digitatum 786	KA<br>H_2O_2	/<br>/	/<br>/	/	-<br>-	-<br>-	ND [c]

[a] MIC: Minimum inhibitory concentration, MFC: Minimum fungicidal concentration, FICI: Fractional Inhibitory Concentration Indices, FFCI: Fractional Fungicidal Concentration Indices. [b] KA was tested up to 12.8 mM. For calculation purpose, 25.6 mM (doubling of 12.8 mM) was used. [c] ND, Not determined (No growth of *P. digitatum* 786).

Table S3. Antifungal chemosensitization of kojic acid (KA; mM) to hydrogen peroxide (H_2O_2; mM) at different temperatures, tested against strains of *Penicillium*: summary of CLSI-based microdilution bioassays (48 h) [a].

28 °C	Compounds	MIC Alone	MIC Combined	FICI	MFC Alone	MFC Combined	FFCI
			Group A				
P. expansum W1	KA<br>H_2O_2	25.6<br>4	3.2<br>2	0.6	25.6 [b]<br>4	12.8<br>2	1.0
P. expansum FR2	KA<br>H_2O_2	25.6<br>4	6.4<br>2	0.8	25.6<br>4	12.8<br>2	1.0
P. expansum W2	KA<br>H_2O_2	25.6<br>4	12.8<br>1	0.8	25.6<br>4	3.2<br>2	0.6
P. expansum FR3	KA<br>H_2O_2	25.6<br>4	6.4<br>2	0.8	25.6<br>4	12.8<br>2	1.0
			Group B				
P. glabrum 766	KA<br>H_2O_2	25.6<br>16	1.6<br>8	0.6	25.6<br>16	12.8<br>8	1.0
P. chrysogenum 824	KA<br>H_2O_2	25.6<br>4	25.6<br>4	2.0	25.6<br>4	25.6<br>4	2.0
P. griseofulvum 2159	KA<br>H_2O_2	25.6<br>4	25.6<br>4	2.0	25.6<br>4	3.2<br>2	0.6
			Group C				
P. italicum 983	KA<br>H_2O_2	25.6<br>2	12.8<br>1	1.0	25.6<br>2	25.6<br>2	2.0
P. digitatum 786	KA<br>H_2O_2	25.6<br>4	12.8<br>2	1.0	25.6<br>4	25.6<br>4	2.0
**35 °C**	**Compounds**	**MIC Alone**	**MIC Combined**	**FICI**	**MFC Alone**	**MFC Combined**	**FFCI**
			Group A				
P. expansum W1	KA<br>H_2O_2	/<br>/	/<br>/	/	25.6<br>1	12.8<br>0.5	1.0
P. expansum FR2	KA<br>H_2O_2	/<br>/	/<br>/	/	25.6<br>2	12.8<br>0.5	0.8
P. expansum W2	KA<br>H_2O_2	/<br>/	/<br>/	/	25.6<br>1	12.8<br>0.5	1.0
P. expansum FR3	KA<br>H_2O_2	/<br>/	/<br>/	/	25.6<br>2	12.8<br>0.5	0.8
			Group B				
P. glabrum 766	KA<br>H_2O_2	25.6<br>4	6.4<br>2	0.8	25.6<br>8	6.4<br>4	0.8
P. chrysogenum 824	KA<br>H_2O_2	25.6<br>1	25.6<br>1	2.0	25.6<br>2	25.6<br>2	2.0
P. griseofulvum 2159	KA<br>H_2O_2	25.6<br>2	3.2<br>1	0.6	25.6<br>2	25.6<br>2	2.0
			Group C				
P. italicum 983	KA<br>H_2O_2	/<br>/	/<br>/	/	25.6<br>1	12.8<br>0.5	1.0
P. digitatum 786	KA<br>H_2O_2	/<br>/	/<br>/	/	25.6<br>0.25	25.6<br>0.25	2.0

Table S3. *Cont.*

45 °C	Compounds	MIC Alone	MIC Combined	FICI	MFC Alone	MFC Combined	FFCI
Group A							
P. expansum W1	KA	/	/	/	25.6	12.8	1.0
	H_2O_2	/	/		0.5	0.25	
P. expansum FR2	KA	/	/	/	25.6	25.6	2.0
	H_2O_2	/	/		0.125	0.125	
P. expansum W2	KA	/	/	/	25.6	12.8	1.0
	H_2O_2	/	/		0.5	0.25	
P. expansum FR3	KA	/	/	/	25.6	12.8	1.0
	H_2O_2	/	/		0.5	0.25	
Group B							
P. glabrum 766	KA	/	/	/	-	-	ND [c]
	H_2O_2	/	/		-	-	
P. chrysogenum 824	KA	/	/	/	-	-	ND [c]
	H_2O_2	/	/		-	-	
P. griseofulvum 2159	KA	/	/	/	25.6	25.6	2.0
	H_2O_2	/	/		2	2	
Group C							
P. italicum 983	KA	/	/	/	-	-	ND [c]
	H_2O_2	/	/		-	-	
P. digitatum 786	KA	/	/	/	-	-	ND [c]
	H_2O_2	/	/		-	-	

[a] MIC: Minimum inhibitory concentration, MFC: Minimum fungicidal concentration, FICI: Fractional Inhibitory Concentration Indices, FFCI: Fractional Fungicidal Concentration Indices. [b] KA was tested up to 12.8 mM. For calculation purpose, 25.6 mM (doubling of 12.8 mM) was used. [c] ND, Not determined (No growth of *Penicillium* strains).
